# Chronic Post-Surgical Pain After Laparoscopic Sleeve Gastrectomy: Is the Opioid-Free Anesthesia Superior? A Cross-Sectional Study

**DOI:** 10.3390/jcm14217721

**Published:** 2025-10-30

**Authors:** Piotr Mieszczański, Marcin Jurczak, Marcin Kołacz, Grzegorz Górniewski, Izabella Godlewska, Paweł Ziemiański, Radosław Cylke, Wojciech Lisik, Janusz Trzebicki

**Affiliations:** 1 1st Department of Anesthesiology and Intensive Care, Medical University of Warsaw, 02-091 Warsaw, Poland; piotr.mieszczanski@wum.edu.pl (P.M.); marcin.jurczak@uckwum.pl (M.J.); janusz.trzebicki@wum.edu.pl (J.T.); 2 Department of Anesthesiology and Intensive Care Education, Medical University of Warsaw, 02-007 Warsaw, Poland; grzegorz.gorniewski@wum.edu.pl; 3 Department of General Surgery and Transplantology, Medical University of Warsaw, 02-006 Warsaw, Poland; izabella.godlewska@uckwum.pl (I.G.); pawel.ziemianski@wum.edu.pl (P.Z.); radoslaw.cylke@wum.edu.pl (R.C.); wojciech.lisik@wum.edu.pl (W.L.)

**Keywords:** chronic post-surgical pain, chronic pain, bariatric surgery, laparoscopic sleeve gastrectomy, opioid-free anesthesia, neuropathic pain

## Abstract

**Background**: Chronic post-surgical pain (CPSP) is a phenomenon that negatively influences patients’ quality of life and well-being. By definition, CPSP is a pain in the surgical area of injury that develops or increases after the operation and persists beyond the healing process. One of the populations that is especially vulnerable to CPSP is patients undergoing bariatric surgery, as obesity, chronic inflammation, pre-existing chronic pain, and severe postoperative pain are its risk factors. Therefore, we conducted a cross-sectional study assessing the prevalence of CPSP in patients undergoing laparoscopic sleeve gastrectomy (LSG). We also aimed to explore the potential influence of the promising opioid-free anesthesia (OFA) technique, assess if the CPSP after LSG had a potential neuropathic component, and additionally, determine whether the bariatric surgery altered chronic pain in this patient population. **Methods**: The study was registered on 11 November 2024, at ClinicalTrials.gov (NCT06686875). A cross-sectional study using e-survey.io was conducted among the patients who underwent LSG 3 months to 5 years earlier. Clinical data were retrieved from the hospital database. **Results**: Of the 135 patients who responded to our e-survey, 4.4% (n = 6, 95% CI 0.9–8%) reported CPSP. None of them had a PAIN DETECT score above 19, which would indicate a neuropathic component. Of the 32 patients who had pre-existing chronic pain, 31 reported a reduction in its intensity, and of the 16 patients on chronic opioid treatment, 10 discontinued opioid therapy. In a subgroup analysis, there was no significant difference in the prevalence of CPSP and long-term opioid therapy between the patients who had OFA and standard anesthesia (*p* > 0.05). **Conclusions**: The main finding of our study is that a minor, yet significant, portion of patients who underwent LSG develop CPSP, and OFA does not alter the risk. LSG appears to reduce pre-existing chronic pain and opioid use.

## 1. Introduction

Chronic post-surgical pain (CPSP) is a complex phenomenon and one of the complications of the surgery, which has a negative influence on the patients’ quality of life and well-being [1]. According to the new, standardized ICD-11 definition [2]. CPSP is pain in the surgical area of injury that develops or increases in intensity after the operation or a tissue injury and persists beyond the healing process, that is, more than 3 months after the triggering event.

Many recognized risk factors may contribute to the development of CPSP: surgical technique, duration, and site, including potential nerve damage, anesthesia mode, especially opioid dosing regimen and regional anesthesia use, severe postoperative pain, patient-related susceptibility, or pre-existing chronic pain [3]. Pain chronification is complex and involves multiple mechanisms at the various levels of nociception [4], including both peripheral, at the surgical trauma site, and central sensitization [5]. One of the populations that is especially vulnerable to these processes is patients with obesity undergoing laparoscopic bariatric surgery, as obesity, chronic inflammation, frequent prevalence of chronic pain, and severe postoperative pain increase the risk of CPSP [3]. Additionally, it may be further exacerbated by ongoing postoperative opioid use after this type of surgery [6].

Considering that over 600,000 bariatric procedures are performed annually [7], the number of patients at risk of CPSP is considerable. Therefore, to investigate this phenomenon, we conducted a study assessing the prevalence of CPSP in patients undergoing the most commonly performed bariatric procedure: laparoscopic sleeve gastrectomy (LSG) [8]. As our primary objective is prevalence estimation, we have designed our research as a cross-sectional study.

LSG has the potential to facilitate considerable excess weight loss, fast recovery, a reduced risk of complications compared to other forms of bariatric surgery, and enhanced quality of life [8]. Nevertheless, certain patients may experience complications such as leaks, bleeding, or gastrointestinal reflux. Additionally, postoperative pain may be considerable.

Opioid-free anesthesia (OFA) is a multimodal anesthesia approach that integrates various analgesic methods without utilizing opioids. Instead, opioids are substituted with adjuncts such as ketamine, dexmedetomidine, lidocaine, and magnesium sulfate [9]. This approach aims to minimize acute opioid exposure. It is particularly promising in bariatric surgery, given the increased opioid sensitivity associated with obesity and the increased risk of postoperative respiratory complications. OFA may reduce postoperative morphine consumption and enhance pain management without compromising hemodynamic stability or increasing sedation [10]. Moreover, the mechanism of action of these co-analgesics indicates that they may influence the chronification of pain in CPSP syndromes by inhibiting NMDA receptors (ketamine and magnesium), α2-adrenergic receptor (α2-AR) agonism, or by preventing inflammatory responses [11].

In our study, we also aimed to explore the potential influence of OFA, to assess if the CPSP after LSG had a potential neuropathic component, and if the bariatric surgery altered chronic pain in this patient population.

## 2. Materials and Methods

### 2.1. Study Design

A cross-sectional study using e-survey.io [12] was conducted between January and April 2025. The study protocol was approved by the Bioethics Committee of the Medical University of Warsaw (AKBE/266/2024), and the study was registered on 11 November 2024, at ClinicalTrials.gov (NCT06686875). The study adhered to the principles outlined in the Declaration of Helsinki, and the manuscript complied with the applicable EQUATOR STROBE guidelines [13]. All the patients provided electronic informed consent to participate in the study.

### 2.2. Patients

In our study, we included patients who underwent primary LSG at the Department of General Surgery and Transplantology of the Medical University of Warsaw between January 2020 and August 2024, and who were aged 18 years or older and provided consent to participate in the study. We excluded patients who underwent revisional procedures, were unable to understand the e-survey, or whom we were unable to contact.

We decided to design a questionnaire as an e-survey as it is an established method in pain research and enables fast and effective data collection, low resource utilization, and fewer errors compared to conventional methods [14].

### 2.3. Data Collection

We collected data from an e-survey sent to patients at least 3 months to 5 years after the operation between December 2024 and April 2025. Baseline clinical data, including preoperative weight, height, BMI, and maximal pain score assessed by the NRS (numeric rating scale) during the first 24 h of the postoperative period and the application of OFA, were collected from hospital documentation. The OFA was used according to the protocol, including dexmedetomidine, lidocaine, ketamine, and magnesium sulfate as outlined in the Supplementary Material S1. As a standard, general anesthesia with multimodal analgesia was used, including simple analgesics, trocar insertion point infiltration with 0.25% bupivacaine, and postoperative analgesia with oxycodone.

### 2.4. E-Survey Questionnaire

The study questionnaire included questions about the current weight (question 1, Q1), the presence of the chronic abdominal pain, which for the first time emerged after the bariatric surgery and persisted for more than 3 months (Q2), the abdominal pain measured at the moment in the NRS scale from 0 to 10 (Q3), the strongest abdominal pain during the past 4 weeks (Q4) and how strong was the abdominal pain during the past 4 weeks on average (Q5), the presence and optionally location of chronic pain before the bariatric surgery (Q15,16), the change in intensity of chronic pain (Q17), the preoperative treatment with opioid analgesics (Q18), and if the patient currently takes opioid analgesics (Q19). Additionally, if patients reported persistent postoperative pain, they were asked to complete the 9-item PAIN DETECT questionnaire (Q6–Q14), adapted in Polish to assess abdominal pain characteristics [15,16]. The English transcription of the questionnaire is attached as Supplementary Material S2.

### 2.5. Study Endpoints

The primary study endpoint was the presence of CPSP after LSG, defined by IASP as new pain at the operation site lasting more than 3 months after surgery, assessed at the time of data collection [2]. The secondary endpoints included abdominal pain intensity and pattern in patients after LSG (Q3–5), whether the abdominal pain in patients after LSG had a probable neuropathic component on assessment utilizing the PAIN DETECT Questionnaire (Q6–14), and the impact of bariatric surgery on chronic pain intensity and opioid use (Q15–19). The cut-off PAIN DETECT Questionnaire indicates a neuropathic pain for scores ≥ 19 points, unclear for scores between 13 and 18, and low probability of a neuropathic component if ≤12 points [15].

### 2.6. Statistical Analysis

All statistical analyses were performed using Statistica software version 13 (TIBCO Software Inc., Palo Alto, CA, USA, 2017). Continuous data were assessed for normality using the Shapiro–Wilk test and compared using either the independent sample *t*-test or the Mann–Whitney U test, as appropriate. Depending on distribution, continuous variables are presented as median (interquartile range, IQR) or mean (standard deviation, SD). Categorical data were assessed for differences between subgroups using Pearson’s χ^2^ analysis or Fisher’s exact test, as appropriate, and presented as numbers and percentages. Statistical significance was set at *p* < 0.05.

## 3. Results

**Figure 1:**
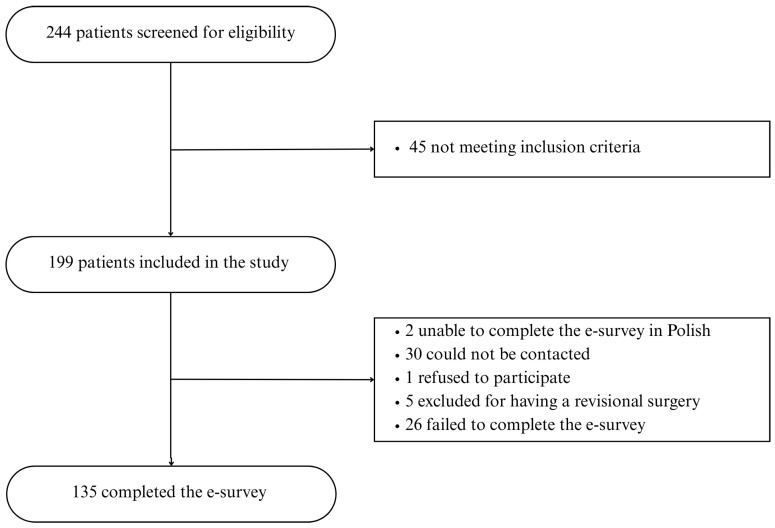
Study flowchart.

244 patients were screened for eligibility, 199 patients met the inclusion criteria, 2 were unable to complete the e-survey in Polish, 30 could not be contacted, 1 refused to participate, 5 were excluded for having a revisional surgery, and 26 failed to complete the e-survey (Figure 1).

The survey was completed by 135 patients who underwent surgery between 2020 and 2024. The average age of the respondents was 44 (range, 21–75), with 70% of them being women (94 individuals). The average initial BMI was 43 (range, 31–65), and the percentage of total weight loss was 28%, with an average BMI of 31 after surgery. The baseline characteristics of the patients are presented in Table 1. The average time from surgery to survey completion was 23 months (range 3–60); anesthesia was performed as OFA in 23 patients (17%).

Six (4.4%, 95% CI 0.9–8%) patients reported pain meeting the IASP criteria for CPSP, and 23 (17%) patients confirmed experiencing any abdominal pain in the last 4 weeks before completing the questionnaire. Of the 6 patients reporting pain meeting CPSP criteria, the average pain during the previous 4 weeks was 4 or more on the NRS scale in 4 of them (67%). The PAIN DETECT score exceeded the 12-point threshold only in 2 patients, above which the neuropathic component is still uncertain but possible [15], indicating that the neuropathic component was minimal or absent. The score did not exceed 10 points in the remaining patients, with a mean of 6.

Among all patients, 32 experienced pain lasting more than 3 months before the procedure, with osteoarticular pain predominating (n = 23), but 8 reported abdominal pain. In 31 cases, the severity of symptoms decreased after surgery, and only one patient reported an increase in pain severity; this individual had previously experienced abdominal pain before the procedure.

A comparative analysis was performed by groups according to the method of anesthesia (OFA vs. general anesthesia with opioids) and the results are presented in the Table 2. No differences were found between the groups in terms of gender, age, baseline and postoperative BMI, maximum NRS values in the perioperative period, and at the time of completing the questionnaire (χ^2^ test for gender, *t*-test or Mann–Whitney U test for the others; *p* > 0.05).

No differences in the incidence of postoperative pain were found between the OFA and opioid anesthesia groups (χ^2^ test or Fisher’s exact test, depending on the number of observations).

In 129 patients without persistent pain, the average body weight before surgery was 122.7 kg, the average BMI was 42.6, the average age was 43.9, and the average EWL was 70%. 17% of them had OFA, and the median maximum postoperative pain intensity was 3.

In 6 patients with persistent pain, the average body weight before surgery was 131.6 kg, the average BMI was 50.1, the average age was 44.9, and the average EWL was 74%. 17% of them had OFA, with a median maximum postoperative pain intensity of 5. Due to the small number of patients, no statistical analysis was performed to compare differences between patients with and without persistent pain.

## 4. Discussion

The main finding of our study is that 4.4% of patients who underwent LSG experienced chronic post-surgical abdominal pain. In these patients, such pain has not been demonstrated to include a neuropathic component and occurred irrespective of the utilization of the OFA technique.

The demonstrated prevalence of CPSP following LSG aligns with the findings of a cross-sectional study conducted by Torensma et al. [17], which reported that 2.3% of patients experienced the onset of new abdominal pain post-surgery. In our study, this proportion was nearly twice as high, potentially attributable to the formulation of the survey question in our research. Additionally, a further distinction is that the majority of participants in their study underwent Roux-en-Y gastric bypass (RYGB); however, the authors observed no significant difference in the prevalence of postoperative chronic pain between the two groups. Furthermore, the conclusions derived from both Torensma’s and our study are inherently limited by their cross-sectional designs.

In their prospective longitudinal study, Chahal-Kummen et al. reported that the prevalence of chronic abdominal pain after LSG was as high as 26.9%, with 12.6% experiencing pain onset after the surgery [18]. Notably, their research indicated that patients suffering from chronic pain exhibited significantly lower quality of life scores. This discrepancy between studies likely stems from our stringent adherence to the ICD-11 CPSP definition, as not all cases of chronic abdominal pain post-LSG satisfy CPSP criteria [2,19]. Furthermore, the authors disclosed a wide array of gastrointestinal symptoms, including gastroesophageal reflux and indigestion, which may exacerbate and potentially contribute to the development of chronic abdominal pain.

A comprehensive retrospective analysis further evidenced the gravity of postoperative pain after bariatric surgery, which revealed that abdominal discomfort constituted 11.14% of Emergency Department admissions within the first year following LSG [20]. However, it is essential to note that their research, which focuses on acute postoperative pain, overlaps with chronic pain, precluding direct comparison with our findings. Nonetheless, the study underscores the influence of pain after LSG on patients’ quality of life.

Although research on CPSP following LSG is limited, this phenomenon has been more widely studied in patients who had RYGB, with reported prevalence rates from 15% to 34% [21,22,23,24], which exceeds the prevalence of CPSP after LSG in both the study by Torensma et al. [17] and our research. However, the findings are only partly generalizable because CPSP depends on procedure-specific risk factors that differ between LSG and RYGB. Additionally, there is variability in how studies define and measure chronic abdominal pain.

Generally, patients who satisfied the CPSP definition criteria in our study did not exhibit an unequivocal neuropathic pain pattern; however, in two cases, a neuropathic component could not be definitively excluded. Several hypotheses have been proposed concerning the origin of CPSP in patients following bariatric surgery, including abdominal cutaneous nerve entrapment syndrome (ACNES) [19,25], adhesions, and dumping syndrome, as well as functional and psychological factors [26]. In our study, the likelihood of a neuropathic component was low, which may have implications for the consideration of treatment options for these patients. Nonetheless, the presence of a neuropathic component was assessed solely through the pain questionnaire, without conducting a clinical examination, which would have provided a more sensitive assessment and potentially yielded different outcomes.

One of the promising methods hypothesized to reduce the incidence of chronic pain after surgery is OFA [27,28], especially as drugs such as ketamine, alpha-2 agonists, or lidocaine may have an opioid-sparing effect, prevent hyperalgesia, and lead to improvements in postoperative pain scores [29], thereby decreasing the prevalence of CPSP [30]. However, the amount of research that could verify this hypothesis is scarce. In a small-group RCT of patients who underwent abdominal hysterectomy, opioid-free anesthesia had no impact on chronic pain incidence [31]. In line with our findings, their study does not provide evidence that adapting OFA as a form of CPSP prophylaxis.

On the contrary, in a comprehensive retrospective study involving a heterogeneous patient cohort and various surgical procedures, increased intraoperative fentanyl administration was correlated with a reduction in chronic pain diagnoses three months post-surgery [32]. Considering obesity as a specific indication for OFA [33], which may have clinically beneficial effects that remain controversial [34], further research is necessary to evaluate the impact of OFA on CPSP prevalence.

Concerning the overall influence of metabolic surgery and weight reduction on the severity of chronic pain, our study corroborates previous reports of improvement, particularly in relation to musculoskeletal pain [21,35]. A decrease in symptoms was observed in our research by the vast majority (96.9%) of patients suffering from chronic pain before surgery, and 8 of 16 patients discontinued their chronic opioid therapy. These findings support the consideration of LSG as a potential method for alleviating specific types of chronic pain in obese patients and for decreasing chronic opioid consumption. Conversely, our results contrast with several studies that indicated continued chronic opioid use following bariatric surgery [6,9,36]. The discrepancies can partially be attributed to cultural differences and healthcare organization models, as well as to the fact that, at our center, patients were not routinely prescribed opioids upon discharge for home use [6,9,36].

The persistent postoperative pain following laparoscopic cholecystectomy was evaluated to be between 3.4% and 7%. Given the reduced trauma to the abdominal wall tissues in comparison to laparotomy, the nature of CPSP after laparoscopy may differ from being purely neuropathic. Both of these assertions align with the findings of our study, despite involving non-obese patients who underwent laparoscopic cholecystectomy [37].

The undetermined cause of chronic abdominal pain following bariatric surgery can be diagnosed and managed through laparoscopy; however, this procedure demonstrates an effectiveness rate of 43%. The remaining 57% of patients with CPSP necessitate long-term medical pain management [38].

Following the exclusion of nociceptive (visceral) and neuropathic etiologies, the nociplastic origin of CPSP should be considered. In our study of 30 patients diagnosed with CPSP, the PAIN DETECT score exceeded the 12-point threshold, indicating the possibility of this type of chronic pain, particularly in cases of chronic inflammation [39]. Regrettably, our study did not diagnose patients from this perspective. This aspect should be considered in future research.

This study has several limitations. Firstly, causality cannot be inferred due to the cross-sectional design. Furthermore, the sample size is relatively small and confined to a single medical institution, which impairs the ability to draw definitive conclusions. Additionally, there are inherent risks of bias related to the electronic survey methodology, such as response bias, wherein patients experiencing pain may be more motivated to respond, and sampling bias, which may occur if individuals face difficulties in participating in the electronic survey. Moreover, the assessment of the potential development of the neuropathic pain component was conducted solely based on a PAIN DETECT scale. In contrast, a more objective diagnosis would necessitate a physical examination to confirm neuropathic pain. Finally, in our study, we performed no sample size estimation, as we made no assumptions concerning the prevalence of CPSP, and we arbitrarily included patients who had surgery up to 5 years before.

In the future, larger, multicenter prospective studies should be conducted to better understand the phenomenon of CPSP in patients after LSG and accurately assess risk factors and their potential prevention.

## 5. Conclusions

Considering the limitations of our study, we observed that less than 5% of patients develop CPSP following LSG, with no confirmed neuropathic component. OFA did not reduce CPSP risk, but bariatric surgery significantly improved pre-existing chronic pain and reduced opioid dependence.

## Figures and Tables

**Table 1:** Baseline characteristics. Categorical variables are expressed as a number and a percentage. Continuous variables are expressed as mean and standard deviation.

Variable	Value (% or SD)
Gender (female)	94 (70%)
Age (years)	44 (21–75)
Weight before (kg)	123.12 (23.86)
Weight after (kg)	88.16 (17.97)
Height (m)	1.69 (0.098)
BMI before (kg/m^2^)	42.93 (6.41)
BMI after (kg/m^2^)	30.78 (5.19)
Average maximal pain intensity after surgery	4.15 (2.34)
Time from surgery (months)	23.18 (16.45)
%EWL	70.23% (24.84)
%TWL	27.85% (9.62)

**Table 2:** Postoperative pain comparison of OFA vs. non-OFA.

Group	Non-OFA	OR (95% CI)	*p* Value (Chi^2^ or Fisher)
Opioid use before surgery	15/112	0.29 (0.04–2.34)	0.24
Present opioid use	6/112	0	0.33
Abdominal pain in the last 4 weeks	18/112	1.45 (0.48–4.41)	0.56
Chronic post-surgical pain	5/112	0.97 (1.1–8.74)	0.73

## Data Availability

The datasets generated during and/or analyzed during the current study are available from the corresponding author upon reasonable request.

## Supplementary Materials

The following supporting information can be downloaded at: https://www.mdpi.com/article/10.3390/jcm14217721/s1, Supplementary Material S1: OFA Protocol; Supplementary Material S2: English transcription of the Study Questionnaire.

## Abbreviations

The following abbreviations are used in this manuscript:

CPSP	chronic post-surgical pain
LSG	laparoscopic sleeve gastrectomy
OFA	opioid-free anesthesia
BMI	body mass index
NRS	numerical rating scale
IASP	International Association for the Study of Pain
EWL	excess weight loss
TWL	total weight loss
SD	standard deviation
