# Supplementary material for: Chronic Post-Surgical Pain After Laparoscopic Sleeve Gastrectomy: Is the Opioid-Free Anesthesia Superior? A Cross-Sectional Study
Source: J Clin Med. 2025 Oct 30;14(21):7721. doi: 10.3390/jcm14217721 (PMC12608722; doi:10.3390/jcm14217721)
Supplement: Supplementary file 1 [file jcm-14-07721-s001.zip › Supplementary Material 1.pdf.zip!/Supplementary Material 1.pdf]

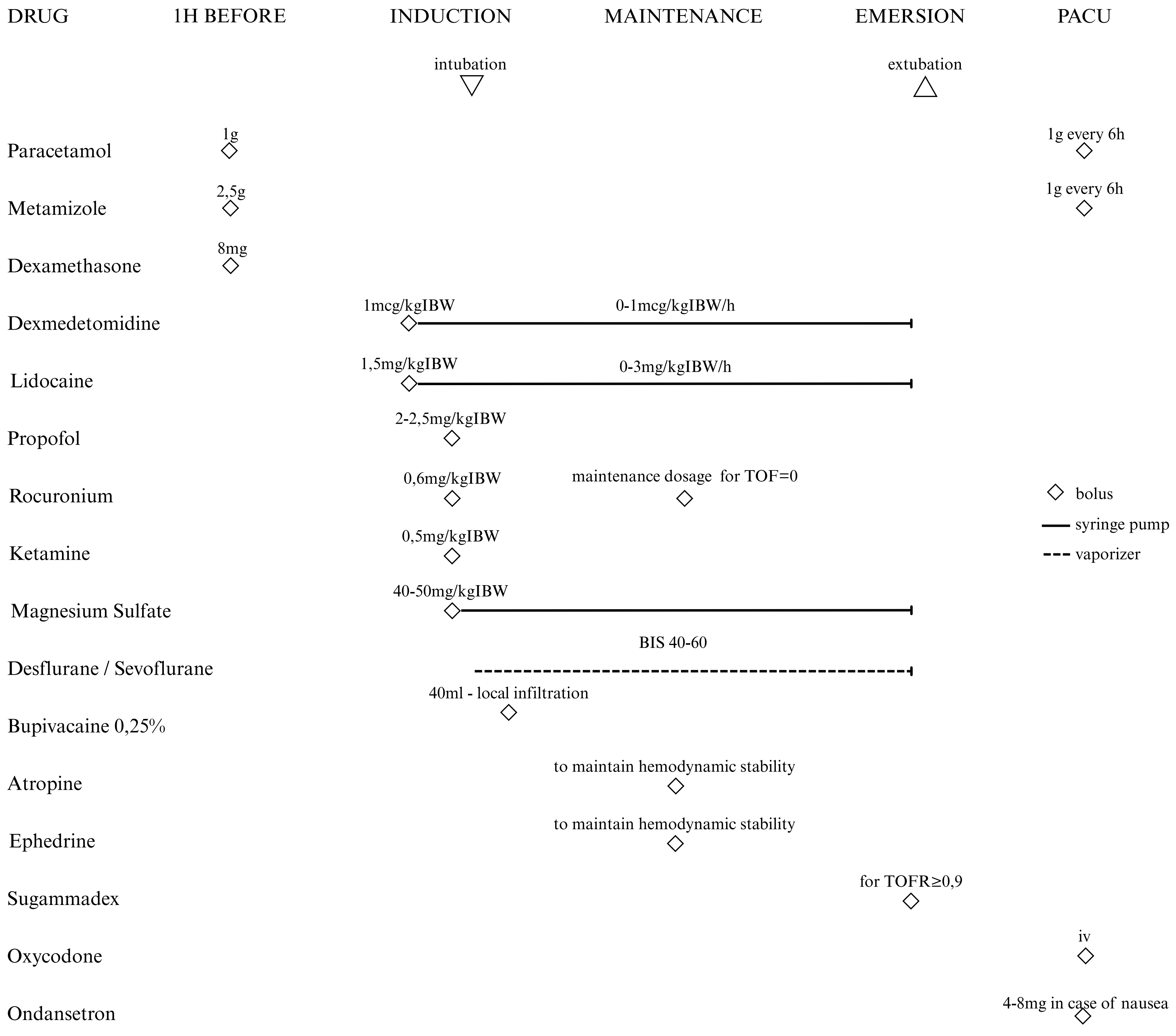
DRUG
1H BEFORE
INDUCTION
MAINTENANCE
EMERSION
PACU
intubation
extubation
Paracetamol
1g
1g every 6h
Metamizole
2,5g
1g every 6h
Dexamethasone
8mg
Dexmedetomidine
1mcg/kgIBW
0-1mcg/kgIBW/h
Lidocaine
1,5mg/kgIBW
0-3mg/kgIBW/h
Propofol
2-2,5mg/kgIBW
Rocuronium
0,6mg/kgIBW
maintenance dosage for TOF=0
Ketamine
0,5mg/kgIBW
Magnesium Sulfate
40-50mg/kgIBW
Desflurane / Sevoflurane
BIS 40-60
Bupivacaine 0,25%
40ml - local infiltration
Atropine
to maintain hemodynamic stability
Ephedrine
to maintain hemodynamic stability
Sugammadex
for TOFR≥0,9
Oxycodone
iv
Ondansetron
4-8mg in case of nausea
bolus
syringe pump
vaporizer
