# Supplementary material for: Chronic Post-Surgical Pain After Laparoscopic Sleeve Gastrectomy: Is the Opioid-Free Anesthesia Superior? A Cross-Sectional Study
Source: J Clin Med. 2025 Oct 30;14(21):7721. doi: 10.3390/jcm14217721 (PMC12608722; doi:10.3390/jcm14217721)
Supplement: Supplementary file 1 [file jcm-14-07721-s001.zip › Supplementary Material 2.pdf.zip!/Supplementary Material 2.pdf]

[Study Questionnaire in Polish with English transcription]

Q1. Jaka jest Pani / Pana aktualna waga?

What is your current weight?

Q2. Czy po operacji pojawił się ból brzucha, który utrzymywał się ponad 3 miesiące?

Did you experience abdominal pain after the surgery that lasted for more than 3 months?

Q3. Jak ocenia Pani / Pan natężenie bólu brzucha w tej chwili?

How would you assess your pain now, at this moment?

Q4. Jak ocenia Pani / Pan najsilniejsze natężenie bólu brzucha w czasie ostatnich 4 tygodni?

How strong was the strongest pain during the past 4 weeks?

Q5. Jak silne było natężenie bólu brzucha w ciągu ostatnich 4 tygodni - przeciętnie?

How strong was the pain during the past 4 weeks on average?

Q6. Proszę zaznaczyć najbardziej odpowiedni wzorzec bólu brzucha (rodzaj bólu).

Mark the picture that best describes the course of your pain:

Persistent pain with slight fluctuations

Persistent pain with pain attacks

Pain attacks without pain between them

Pain attacks with pain between them

Q7. Czy ból brzucha ma charakter promieniowania?

Does your abdominal pain radiate to other regions of your body?

Q8. Czy odczuwa Pani / Pan palenie (np. pokrzywkę) w okolicy dolegliwości bólowych brzucha?

Do you suffer from a burning sensation (e.g., stinging nettles) in the area where you experience abdominal pain?

Q9. Czy odczuwa Pani / Pan mrowienie, igiełki, przechodzenie prądu w okolicy dolegliwości bólowych brzucha?

Do you have a tingling or prickling sensation in the area where you experience abdominal pain?

Q10. Czy delikatny dotyk w okolicy brzucha jest bolesny?

Is light touching in abdominal area painful?

Q11. Czy miewa Pani / Pan niespodziewane / nagłe strzały prądu w okolicy dolegliwości bólowych brzucha?

Do you have sudden electric shock-like pain attacks in the area where you experience abdominal pain?

Q12. Czy zdarza się, że zimno lub gorąco otoczenia (np. kąpiel) wywołuje ból w okolicy brzucha?

Is cold or heat (e.g., bath water) in abdominal area occasionally painful?

Q13. Czy odczuwa Pani / Pan odrętwienie w okolicy dolegliwości bólowych brzucha?

Do you suffer from a sensation of numbness in the area where you experience abdominal pain?

Q14. Czy delikatny nacisk w okolicy brzucha wywołuje ból?

Does slight pressure in abdominal area trigger pain?

Q15. Czy przed operacją odczuwała Pani / Pan jakiś ból przewlekły?

Did you experience any chronic pain before the surgery?

Q16. Gdzie zlokalizowany był ból przewlekły odczuwany przed operacją?

Where was the chronic pain you experienced before the surgery located?

Q17. Czy po operacji natężenie tego bólu przewlekłego się zmieniło?

Has the intensity of this chronic pain changed after the surgery?

Q18. Czy przed operacją przyjmowała Pani/przyjmował Pan opioidowe leki przeciwbólowe?

Were you taking opioid pain medications before the surgery?

Q19. Czy obecnie przyjmuje Pani/ Pan opioidowe leki przeciwbólowe?

Are you currently taking opioid pain medications?
